# A Measurement Method for Interfaces in Multiphase Mixed Media Based on Ultrasonic Transmission

**DOI:** 10.3390/s26092683

**Published:** 2026-04-26

**Authors:** Bin Yu, Hongbo Liao, Fenglong Yin, Ji’ang Zhao, Yunyi Tang, Yukun Fu, Mingrui Xie, Dong Han

**Affiliations:** 1 Northwest Institute of Nuclear Technology, Xi’an 710024, China; yubin17a@nudt.edu.cn (B.Y.); 13899090352@163.com (F.Y.); ji_ang001@126.com (J.Z.); tangyunyi99@163.com (Y.T.); fykcug2025@163.com (Y.F.); 2 State Key Laboratory of Fluid Power and Mechatronic Systems, School of Mechanical Engineering, Zhejiang University, Hangzhou 310058, China; 12225107@zju.edu.cn (M.X.); dong_han@zju.edu.cn (D.H.)

**Keywords:** ultrasonic transmission, multiphase mixed media, interface measurement, acoustic field characterization, liquid level detection

## Abstract

This paper addresses the challenge of accurately measuring liquid level interfaces in multiphase mixed media by proposing a detection method based on ultrasonic transmission. First, a mathematical model of the ultrasonic measurement system was established, and the acoustic field characteristics of transducers with different frequencies and diameters in slurry were simulated and analyzed to determine the optimal excitation frequency and probe diameter. On this basis, an echo sound pressure calculation model based on the side-incidence method was constructed, and a formula for calculating the liquid level interface height was derived. Finally, an experimental test platform with a multi-layer steel container was built to measure the propagation velocity, attenuation coefficient, and acoustic impedance coefficient of ultrasound in the slurry, verifying the feasibility of the liquid level interface measurement method.

## 1. Introduction

Accurate liquid level measurement constitutes a core technology in modern industrial process control, safety monitoring, and resource management, with extensive applications across the chemical, petroleum, pharmaceutical, and water treatment sectors. Particularly within sealed containers or complex structures containing multiphase mixed media—such as slurries, emulsions, or stratified fluids—the real-time and precise acquisition of liquid-level interface information is of paramount importance for preventing leaks, avoiding overflows, optimizing reaction processes, and ensuring personnel and environmental safety. However, multiphase mixed media are typically characterized by complex composition, inhomogeneous acoustic properties, opacity, and strong acoustic attenuation, which pose significant challenges to conventional liquid level measurement techniques.

To meet measurement requirements across diverse application scenarios, a wide variety of liquid level sensing technologies have been developed in academia and industry. These technologies can be broadly categorized into contact and non-contact types based on their operating principles. Traditional contact methods, such as float-type, hydrostatic pressure, and capacitive sensors, although simple in structure, suffer from issues like susceptibility to contamination, difficult maintenance, or significant measurement inaccuracy due to medium properties when dealing with corrosive, highly viscous, or scaling-prone media [1,2]. Non-contact methods are favored for their avoidance of direct contact with the medium, with ultrasound, optical, and microwave technologies being prominent representatives.

Ultrasonic technology has become one of the most widely researched and applied non-contact liquid level measurement methods due to its strong adaptability, high reliability, and relatively low cost. Conventional pulse-echo methods calculate liquid level by measuring the time-of-flight of an ultrasonic wave reflected from the liquid surface. However, they often fail in scenarios involving low liquid levels, highly attenuating media, or containers with complex internal structures due to weak signals and multiple reflection interference [3]. Numerous improvements have been proposed to enhance performance. For instance, the use of guided wave modes (e.g., longitudinal, torsional, and flexural modes) propagating in waveguides leverages the differential sensitivity of various modes to fluid immersion for measurement, improving remote monitoring capabilities in harsh environments [4,5,6,7]. Miniaturized sensors based on Capacitive Micromachined Ultrasonic Transducers (CMUT) offer high sensitivity, enabling detection of minute liquid level changes and even leak detection in small-volume reservoirs [8]. For the automatic detection of liquid–liquid interfaces, self-calibrating ultrasonic sensors combined with advanced signal processing algorithms enable robust measurement even without prior knowledge of the media’s acoustic characteristics [9]. Furthermore, other innovative acoustic methods continue to emerge, such as the transverse pulse train technique propagating in a vertical hollow wire, which achieves high-precision measurement by simultaneously detecting two echo signals from the liquid surface and the wire end, with the interface echo method demonstrating exceptionally low error [10]. Other research avenues include techniques based on Lamb waves [11,12], resonance [3], synthetic aperture focusing [13], or the integration of machine learning for signal processing [14,15], all aimed at enhancing detection accuracy and robustness for low fill levels, dynamic levels, or liquid levels within pipes. Magnetostrictive sensors utilize the Wiedemann effect and time-frequency analysis techniques like wavelet transform for high-precision measurement [16]. Nevertheless, for the high-density, highly attenuating multiphase slurry of interest in this study, ultrasonic energy attenuates rapidly, and traditional top-incidence vertical echo methods often fail to obtain effective liquid surface reflection signals, limiting the direct application of these approaches.

Optical sensing technology represents another significant research direction. Fiber Bragg Grating (FBG) sensors measure liquid level indirectly by sensing hydrostatic pressure changes or temperature distribution, with designs incorporating multiple sensors or reference gratings to compensate for temperature cross-sensitivity [17,18]. Polymer Optical Fiber (POF) sensors demonstrate potential in special environments due to their inherent safety, immunity to electromagnetic interference, chemical corrosion resistance, and flexibility, operating primarily on intensity or wavelength modulation principles [19]. Fiber-optic sensors based on a Michelson interferometer structure achieve millimeter or even sub-millimeter resolution by demodulating the amplitude of the spectral Fast Fourier Transform within a specific wavelength range [20]. Additionally, some novel optical methods, such as using reciprocally offset LEDs and phototransistors to determine level changes by detecting optical axis shifts, claim insensitivity to the optical properties of the liquid itself [21]. However, optical methods still commonly face the challenge of signal failure due to obstructed light paths or fouled probes in multiphase, opaque, or contaminating media.

Microwave and radio frequency technologies measure liquid level based on the influence of the medium on electromagnetic wave properties. Frequency Domain Reflectometry (FDR) and microwave interferometry enable highly sensitive detection of minute liquid level changes and can identify stratified liquid interfaces [22,23]. Sensors based on microstrip transmission lines [24] or hollow coaxial cable resonators [22] also exhibit excellent performance. Moreover, the design of wireless, passive sensors based on LC resonant circuits offers a novel approach for level measurement in harsh environments (e.g., corrosive, cryogenic fluids) [25]. Although microwave technology performs well with non-conductive media, its penetration depth and measurement accuracy significantly degrade in conductive slurries or media with high dielectric loss.

Beyond sensors based on a single physical principle, multi-sensor data fusion and intelligent processing have become key trends for enhancing system adaptability, accuracy, and functionality. By fusing information from various sensors such as capacitive, ultrasonic, and pressure sensors, and combining it with machine learning algorithms like Support Vector Machine (SVM), simultaneous accurate prediction of liquid level and medium concentration (e.g., sugar content) can be achieved, overcoming the limitations of single sensors being affected by medium type [26,27]. Machine learning is also directly applied to process temperature data from FBG arrays or acoustic signals in oil wells for level estimation via classification and regression [15,18]. Some innovative designs, such as cost-effective, high-resolution water level monitoring systems combining load cells with floating bodies, also demonstrate the effectiveness of simple physical principles in specific applications [28].

In summary, although existing liquid level measurement technologies have made significant progress and demonstrate excellent performance under specific conditions, they exhibit clear limitations when applied to the specific scenario addressed in this study: sealed containers with complex internal structures filled with highly attenuating and opaque multiphase mixed slurries. Top-incidence vertical ultrasonic echo methods suffer from weak or non-existent signals; optical methods are easily obstructed by the medium; and microwave technology underperforms in conductive slurries. Therefore, developing a non-invasive measurement method capable of penetrating thick walls, adapting to highly attenuating media, and accurately capturing liquid level interface changes holds significant theoretical value and engineering importance.

To address the aforementioned challenges, this paper proposes a liquid level interface measurement method for multiphase mixed media based on side-incidence ultrasonic transmission energy analysis. The core concept of this method is as follows: Ultrasonic probes are fixed on the external sidewall of the container. The position of the liquid level relative to the probe’s acoustic radiation zone is detected by analyzing the energy variation in the echo signals resulting from multiple reflections of the ultrasonic wave within the multilayer structure formed by the container wall and the internal medium. As the slurry level gradually immerses the probe’s detection zone, the improved acoustic impedance match between the slurry and the container wall (compared to the gas–wall interface) causes more ultrasonic energy to transmit into the slurry, leading to a significant decrease in the received echo sound pressure energy. By establishing a system mathematical model, optimizing probe parameters, and constructing an echo sound pressure calculation model based on the detection energy zone, the liquid level height can be derived, enabling non-invasive and precise interface localization.

In contrast to medical ultrasound or sonar applications where the propagation medium typically exhibits low acoustic attenuation, the slurry in this study has a high attenuation coefficient and is opaque, rendering optical methods infeasible and conventional top-incidence ultrasonic echo methods ineffective. Furthermore, the container walls are thick (8–20 mm) and have complex internal structures, which further complicates signal interpretation. Direct quantitative comparison with other techniques (e.g., fiber-optic sensors, CMUTs, or microwave methods) is not meaningful under these conditions: fiber-optic sensors are blocked by opacity, CMUTs require thin acoustic windows, and microwave methods suffer from severe signal loss in conductive slurries. In contrast, the proposed side-incidence transmission method, which relies on echo energy changes from multiple reflections within the container wall, is specifically tailored to overcome these challenges.

The main contributions of this work are as follows: (1) establishment of a comprehensive mathematical model for the entire ultrasonic measurement system, encompassing the excitation, propagation, and reception chain; (2) determination of the optimal probe frequency (1 MHz) and diameter (20 mm) for the specified slurry medium through acoustic field simulation and optimization; (3) proposal of a liquid level interface detection algorithm based on side-incidence multiple reflection superposition and derivation of the liquid level height calculation formula; and (4) development of an experimental platform, measurement of key ultrasonic propagation parameters in the slurry (sound velocity ~2372 m/s, attenuation coefficient ~15.1 dB/m, acoustic impedance ~4.1 × 10^5^ g/(cm^2^·s)), and experimental verification of a measurement accuracy within ±2 mm for the liquid level interface under different wall thickness conditions (8 mm and 20 mm). These characteristics make the method particularly suitable for industrial applications involving sealed containers with multiphase mixed media.

The remainder of this paper is organized as follows: Section 2 details the system modeling and transducer parameter optimization via simulation. Section 3 elaborates on the interface detection algorithm based on side-incidence echo energy analysis. Section 4 presents the experimental platform setup, key parameter measurements, and validation results for liquid level interface detection. Finally, Section 5 concludes the paper and suggests directions for future work.

## 2. Overall System Architecture and Mathematical Modeling

This section establishes a mathematical model of the ultrasonic liquid-level interface measurement system, which primarily consists of key components such as the transmitter/receiver circuit, connecting cables, ultrasonic transducers, and the measured medium. Based on this, a comprehensive overall model of the measurement system was constructed.

**Figure 1:**
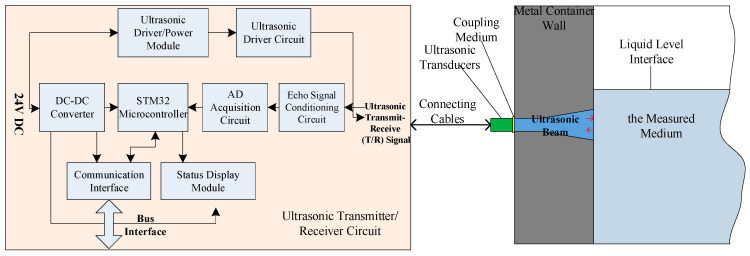
System composition of the ultrasonic liquid-level interface measurement system.

The structure of the ultrasonic liquid-level interface measurement system is illustrated in Figure 1. It primarily consists of an ultrasonic transmitter/receiver circuit, connecting cables, ultrasonic transducers, a coupling medium, a metal container wall, and the measured medium. The establishment of the overall system model is based on mathematical representations of the ultrasonic transmitter/receiver circuit, the transducers, and the measured medium, with the input and output physical signals of each stage serving as the linkages, thereby enabling the integrated construction of the complete measurement system model.

Based on the aforementioned system composition, the model can be divided into three main modules: the ultrasonic transmission system, the ultrasonic reception system, and the measured medium. The integrated overall system model was constructed on the basis of the mathematical models of these subsystems.

### 2.1. Ultrasonic Transmission System Model

**Figure 2:**
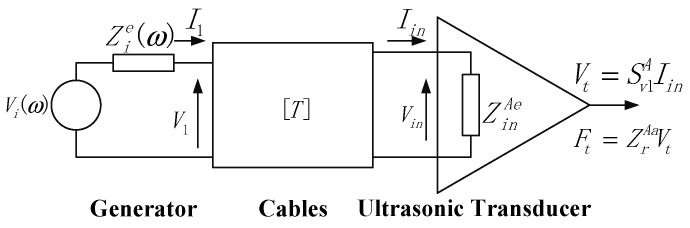
The model of the ultrasonic transmission system.

The model of the ultrasonic transmission system is shown in Figure 2. It consists of three main components: an ultrasonic generator, connecting cables, and a transmission transducer. The entire transmission system is modeled as a single-input single-output (SISO) time-invariant system, with its transfer function denoted as *T_G_*(*ω*). In the following equations, all frequency-dependent parameters (such as electrical impedance *Z*, sensitivity *S*, radiation impedance *Z_rad_*, and wave number *k*) are understood to be functions of *ω*, even when not explicitly written for brevity. The symbol *ω* on the left-hand side of transfer functions indicates that these are frequency-domain representations.

The transfer function *T_G_*(*ω*) can be expressed as:

(1) $$T_{G}(\omega )=\frac{F_{t}\left(\omega \right)}{V_{i}\left(\omega \right)}=\frac{Z_{r}^{Aa}S_{v1}^{A}}{\left(Z_{in}^{Ae}T_{11}^{C}+T_{12}^{C}\right)+\left(Z_{in}^{Ae}T_{21}^{C}+T_{22}^{C}\right)Z_{i}^{e}},$$

where $T_{11}^{C}$, $T_{12}^{C}$, $T_{21}^{C}$, and $T_{22}^{C}$ represent the transmission matrix parameters of the connecting cable at the transmitter side; $Z_{in}^{AE}$ denotes the input electrical impedance of the transmitting ultrasonic transducer; $S_{v1}^{A}$ represents the sensitivity of the transmitting transducer; $Z_{r}^{Aa}$ indicates the acoustic radiation impedance of the transmitting transducer; and $Z_{i}^{e}$ corresponds to the output electrical impedance of the ultrasonic generator.

### 2.2. Ultrasonic Reception System Model

**Figure 3:**
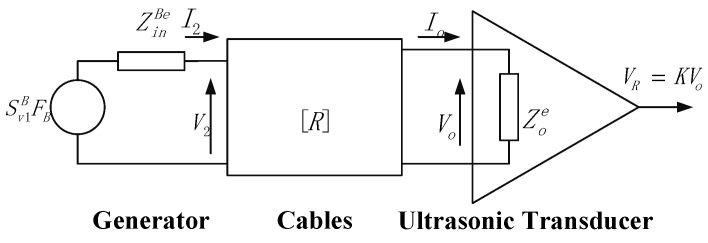
The model of the ultrasonic reception system.

The model of the ultrasonic reception system is illustrated in Figure 3. In the reception system, the incident acoustic pressure is converted into an electrical signal by the receiving transducer. This signal is then transmitted via connecting cables to the receiver circuit, where it undergoes amplification processing to produce a final output voltage.

The transfer function of the ultrasonic reception system, denoted as *R*(*ω*), is given by:

(2) $$R_{G}\left(\omega \right)=\frac{V_{R}\left(\omega \right)}{F_{B}\left(\omega \right)}=\frac{KZ_{o}^{e}S_{v1}^{B}}{\left(Z_{in}^{Be}R_{11}^{C}+R_{12}^{C}\right)+\left(Z_{in}^{Be}R_{21}^{C}+R_{22}^{C}\right)Z_{o}^{e}},$$

where $R_{11}^{C}$, $R_{12}^{C}$, $R_{21}^{C}$, $R_{22}^{C}$ represent the transmission matrix parameters of the connecting cable at the receiver side; $Z_{in}^{BE}$ denotes the input electrical impedance of the receiving ultrasonic transducer; $S_{v1}^{B}$ represents the sensitivity of the receiving transducer; and $Z_{o}^{e}$ corresponds to the input electrical impedance of the ultrasonic receiver.

### 2.3. Wave Propagation Model in the Measured Medium

In a complete ultrasonic measurement system, the acoustic transfer function $T_{A}\left(\omega \right)$ for wave propagation through the elastic medium is defined as:

(3) $$T_{A}\left(\omega \right)=\frac{F_{B}\left(\omega \right)}{F_{t}\left(\omega \right)}=2R_{12}\exp\left(2iK_{p1}D\right)\left[1-exp\left(\frac{K_{p1}r^{2}}{2D}\right)\left\{J_{0}\left(\frac{K_{p1}r^{2}}{2D}\right)-iJ_{1}\left(\frac{K_{p1}r^{2}}{2D}\right)\right\}\right],$$

where $R_{12}$ is the plane wave reflection coefficient at the interface; *D* represents the distance between the centers of the transmitting and receiving transducers; *J*_0_ and *J*_1_ denote the zero-order and first-order Bessel functions, respectively; *r* corresponds to the effective radius of the transducer; and *K_p_*_1_ indicates the acoustic attenuation coefficient of the medium.

Based on the above analysis, the complete mathematical model of the ultrasonic measurement system can be expressed as follows:

(4) $$\frac{V_{R}\left(\omega \right)}{V_{i}\left(\omega \right)}=T_{G}\left(\omega \right)R_{G}\left(\omega \right)T_{A}\left(\omega \right).$$

### 2.4. Simulation Analysis and Optimization of Transducer Parameters

The initial values of the model parameters for the ultrasonic liquid-level interface measurement system are listed in Table 1. With the transducer diameter held constant, the acoustic field characteristics of ultrasound in the slurry were analyzed at excitation frequencies of 500 kHz, 1 MHz, and 2 MHz. With the excitation frequency held constant, the acoustic field characteristics were analyzed for transducer diameters of 10 mm and 20 mm.

When only the transducer frequency was varied while keeping other system parameters unchanged, the following trend was observed. As the transducer frequency increased, the propagation attenuation of ultrasound in the slurry became larger, while the energy in the longitudinal near-field region became more concentrated. Although low-frequency transducers offer a longer propagation distance in the slurry, their energy is relatively more dispersed compared to high-frequency ones, making them less than optimal for a liquid-level detection method that relies on evaluating the near-field energy magnitude. Furthermore, increasing the frequency not only enhances the near-field energy but also imposes higher demands on the transmitting/receiving and signal processing circuits. Therefore, from the perspective of implementation cost, ultra-high frequency ultrasonic transducers were not considered. Based on the above analysis, the 1 MHz ultrasonic transducer represents a relatively optimal choice.

When only the transducer diameter was varied while keeping other system parameters unchanged, a larger transducer diameter resulted in higher emitted energy and consequently higher reflected energy at the inner container wall, which is beneficial for the liquid-level detection method based on near-field energy evaluation. However, the transducer diameter should not be excessively large, as an oversized diameter would compromise the positioning accuracy of the liquid-level interface within the transducer’s diameter range. Based on the above analysis, the transducer diameter was determined to be 20 mm.

## 3. Interface Detection Algorithm Based on Side-Incidence Echo Energy

This section analyzes the key characteristics of the echo signals from the liquid-level interface under the side-incidence configuration. A computational model for the echo sound pressure, based on the probe’s detection energy zone, is established. Furthermore, the formula for calculating the liquid-level height is derived accordingly.

### 3.1. Principle of Side-Incidence Interface Detection

**Figure 4:**
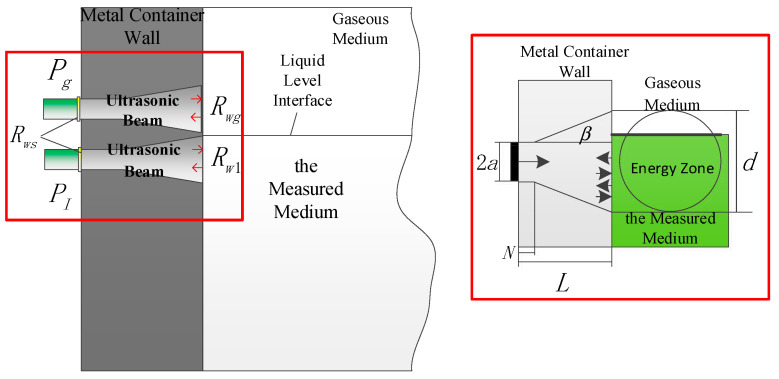
Schematic diagram of the basic principle for ultrasonic liquid-level interface detection. *R_wg_* is the sound pressure reflection coefficient at the inner wall above the liquid level, *R_w_*_1_ is the coefficient below the liquid level, and *R_ws_* is the reflection coefficient between the transducer and the outer wall.

Owing to the complex internal structure of the container under test, measuring the liquid-level interface using ultrasonic echo signals from the top or bottom is not feasible. Consequently, this study employs a method wherein ultrasonic probes are installed at fixed positions on the sidewall of the container. The interface position is determined by detecting the energy difference in the echo signals caused by the presence or absence of the liquid. As illustrated in Figure 4, the transmission and reflection behaviors of the ultrasonic beam differ significantly between the areas above and below the liquid-level interface at the container’s inner wall. As the slurry gradually rises from the bottom, starting to enter the transducer’s detection zone until it is completely covered, the echo sound pressure energy received by the receiving transducer undergoes a distinct variation. The precise location of the liquid-level interface can be determined by calculating this energy difference.

### 3.2. Calculation Model in the Detection Energy Zone

**Figure 5:**
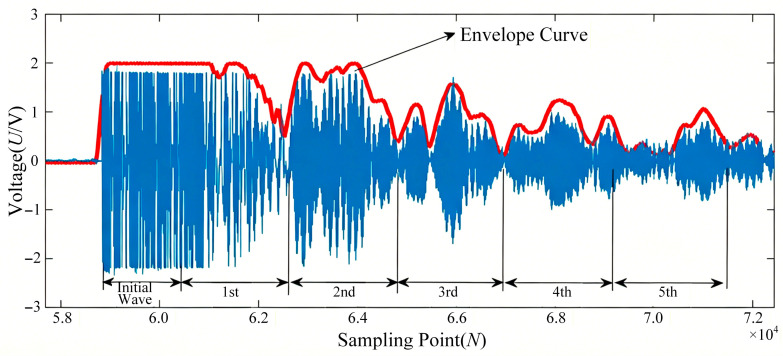
Echo sound pressure distribution within the container wall after multiple reflections.

Based on the aforementioned liquid-level interface detection principle, the echo sound pressure was modeled and calculated using Kirchhoff’s approximation theory. The distribution of the echo sound pressure within the container wall after multiple reflections is illustrated in Figure 5.

Given the container wall thickness *L*, the transducer radius *a*, the diameter of the detection energy zone *d*, and the initial incident sound pressure *P*_0_, the average sound pressure of the first reflection echo at the inner container wall, denoted as ${\bar{P}}_{r}$, can be expressed as:

(5) $${\bar{P}}_{r}=P_{0}e^{-\alpha L}R_{w}\left(4a^{2}/d^{2}\right),$$

where *R_w_* represents the ultrasonic reflection coefficient at the inner container wall, which is calculated by:

(6) $$R_{w}=\frac{\rho_{2}c_{2}cos\theta_{i}-\rho_{1}c_{1}cos\theta_{b}}{\rho_{2}c_{2}cos\theta_{i}+\rho_{1}c_{1}cos\theta_{b}},$$

where *ρ*_1_ and *ρ*_2_ are the densities of the two media, respectively, *c*_1_ and *c*_1_ represent the ultrasonic propagation velocities in the corresponding media, *θ_i_* denotes the angle of incidence, and *θ_b_* is the angle of reflection.

When the acoustic beam is reflected by the outer container wall and arrives at the inner wall for the second time, the sound pressure, denoted as *P_L_*_2_, can be expressed as:

(7) $$P_{L2}={\bar{P}}_{r}R_{ws}e^{-\alpha L}.$$

After being reflected at the inner wall, the sound pressure upon reaching the outer container wall for the second time, denoted as $P_{L2}^{\prime }$, is given by:

(8) $$P_{L2}^{\prime }={\bar{P}}_{r}r_{s}R_{ws}R_{wg}e^{-2\alpha L}+{\bar{P}}_{r}\left(1-r_{s}\right)R_{ws}R_{w1}e^{-2\alpha L},$$

where *r_s_* represents the ratio of the area exceeding the liquid level to the total area of the detection energy zone. By analogy, the sound pressure of the ultrasonic wave upon reaching the outer container wall and being received by the transducer for the *n*-th time, denoted as *P_n_*, can be expressed as:

(9) $$\sum P_{n}={\bar{P}}_{r}(r_{s}\sum_{i=1}^{n}R_{ws}^{i-1}R_{wg}^{i}e^{-\left(2i-1\right)\alpha L}+\left(1-r_{s}\right)\sum_{i=1}^{n}R_{ws}^{i-1}R_{w1}^{i}e^{-\left(2i-1\right)\alpha L}).$$

**Figure 6:**
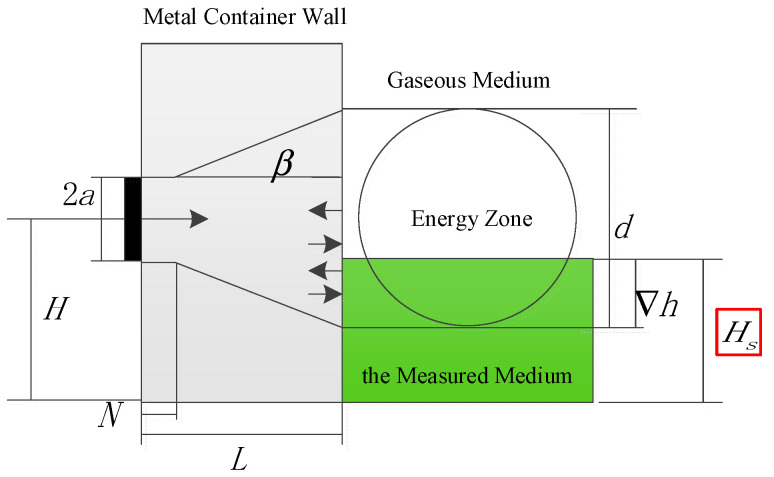
Schematic diagram of the principle for determining the liquid-level interface.

In summary, the total echo sound pressure received by the ultrasonic transducer at the outer container wall is the superposition of the sound pressures from multiple echoes generated by the acoustic beam undergoing successive reflections within the wall structure. Based on this multiple-reflection superposition model, the precise position of the liquid-level interface relative to the transducer’s detection energy zone can be calculated. The underlying calculation principle is illustrated in Figure 6.

The diameter of the detection energy zone *d* depends on the near-field length *N*, and it can be calculated by the following formula:

(10) $$\left\{\begin{array}{c} d=2a\left(L\leq N\right)\\ d=2\left[a+\left(L-N\right)\tan\left(\beta \right)\right](L>N) \end{array}\right..$$

Once the diameter of the detection energy zone *d* is known, the liquid-level interface height *H_s_* is calculated using the following formula:

(11) $$H_{s}=H-\frac{d}{2}+\nabla h,$$

where *H* represents the installation height of the ultrasonic probe, and ∇*h* denotes the relative height of the liquid-level interface within the probe’s detection energy zone. Where the relative height ∇*h* is calculated by:

(12) $$\nabla h=\frac{d-d\left(\cos\left(\pi \left(\frac{\sum P_{i}-\sum P_{0}}{\sum P_{n}-\sum P_{0}}\right)\right)\right)}{2},$$

where $\sum P_{n}$ denotes the total sound pressure value of the echo signal when the liquid level is at the highest position within the detection energy zone, $\sum P_{0}$ represents the total sound pressure value when the liquid level is at the lowest position within the detection energy zone, and $\sum P_{i}$ corresponds to the total sound pressure value at the current liquid level within the detection energy zone.

### 3.3. Theoretical Analysis of Echo Signal Variation

**Figure 7:**
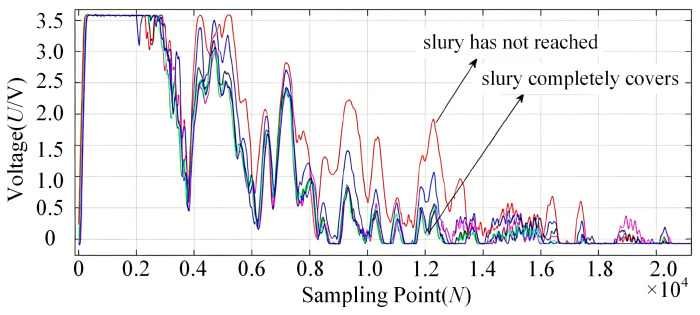
Variation in the echo signal during the slurry injection process.

The variation in echo sound pressure during the slurry injection process is shown in Figure 7. In the initial stage of injection, when the slurry has not yet reached the probe’s detection zone, the inner wall of the container is in contact with the gaseous medium. The acoustic reflection coefficient under this gas–wall interface condition is relatively high, resulting in high echo signal energy. As the slurry front rises and enters the detection zone, the inner wall comes into contact with the slurry. The acoustic reflection coefficient at this slurry–wall interface is significantly lower than that of the gas–wall interface due to the substantial difference in acoustic impedance between the slurry and the gas, leading to a noticeable decrease in echo signal energy. This reduction occurs because a portion of the acoustic energy is transmitted through the container wall into the slurry.

It should be noted that the experimental validation in this study was conducted primarily during the slurry filling (rising liquid level) process. In practical applications where the liquid level may also descend, residual slurry may adhere to the inner wall of the container, forming a thin film that alters the acoustic boundary condition. This adhered layer can maintain a degree of acoustic impedance matching even after the bulk liquid level has fallen below the detection zone, potentially leading to a hysteresis effect in the echo energy response. Consequently, the detection accuracy during the draining process may differ from that during filling. For applications requiring precise measurement in both filling and draining cycles, future work should focus on developing adaptive threshold algorithms or integrating cleaning mechanisms to mitigate the influence of wall adhesion. This aspect represents an important direction for further enhancing the robustness and practical applicability of the proposed method.

The proposed side-incidence interface detection method fundamentally relies on the difference in acoustic reflection characteristics at the container inner wall when the adjacent medium changes from gas to slurry. When the inner wall is in contact with gas, the large acoustic impedance mismatch between the gas and the container wall results in a high reflection coefficient, meaning that most of the acoustic energy is reflected back and received by the transducer. In contrast, when the slurry contacts the inner wall, the acoustic impedance mismatch is considerably smaller, leading to a lower reflection coefficient and a corresponding reduction in the received echo energy.

In practical applications, the concentration or composition of the slurry may vary, which in turn affects its acoustic properties. However, the detection principle does not depend on the exact value of the slurry’s acoustic impedance, but rather on the presence of a sufficient impedance contrast between the slurry and the gas. For typical multiphase slurries, their acoustic impedance remains several orders of magnitude higher than that of gas, regardless of moderate variations in concentration or composition. Consequently, the reflection coefficient at the slurry–wall interface remains significantly lower than that at the gas–wall interface, ensuring that the echo energy drop upon liquid level entry is consistently distinguishable.

Therefore, the proposed method is expected to remain effective across a reasonable range of slurry formulations. It should be noted, however, that extreme variations in slurry properties—such as a drastic reduction in acoustic impedance or the introduction of highly attenuating components—may affect the absolute echo energy amplitude and potentially influence detection sensitivity. In such cases, system recalibration or adaptive threshold adjustment may be required, which is identified as a direction for future research.

The echo signals were processed according to the aforementioned sound pressure calculation formulas. The received ultrasonic signal is sampled at 2 MHz with 14-bit resolution. The integration time window from 0.05 ms to 1.75 ms was selected based on the geometric parameters of the experimental setup and the propagation characteristics of ultrasound. The lower bound of 0.05 ms is set to exclude the high-amplitude initial excitation pulse and the near-field interference that occurs immediately after transmission, ensuring that the integrated signal primarily reflects the energy from the first and subsequent echoes generated by multiple reflections within the container wall and the slurry. The upper bound of 1.75 ms is determined to encompass the major multiple reflection events. Given the container wall thickness (8–20 mm), the sound velocities in steel (approximately 2540 m/s) and in the slurry (approximately 2372 m/s), and the transducer installation positions, the time required for the ultrasonic wave to propagate through the structure and undergo multiple reflections is estimated to be within this range. This window thus captures the critical portion of the echo signal that carries information about the liquid-level interface while minimizing noise and irrelevant interference.

The received ultrasonic signal is sampled at 2 MHz with 14-bit resolution. A time window from 0.05 ms to 1.75 ms is applied to exclude the initial excitation pulse and capture the main multiple reflections (up to the 10th echo, beyond which the amplitude falls below the noise floor). The signal is then full-wave rectified and integrated over the window to obtain a scalar value representing the total echo energy. This integration is performed for each measurement cycle. To compute the total sound pressure *P_total_*, the infinite series is truncated to *n* = 10 terms, as higher-order contributions are negligible (attenuation reduces them to <1% of the first echo). The reflection coefficients *R_wg_*, *R_w_*_1_ and *R_ws_* are pre-calibrated using the measured acoustic impedances. The liquid-level height is then derived using Equations (10)–(12) with the integrated echo energy as input.

**Figure 8:**
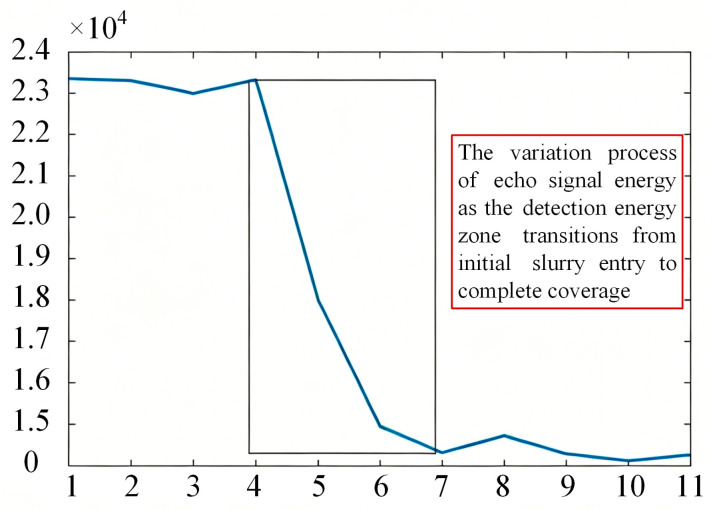
Integral values of multiple echoes during the slurry injection process.

The integral values from each measurement were plotted as a curve, as shown in Figure 8. Analysis reveals that when the slurry has not reached the probe’s detection zone, the integral values of the multiple echoes exhibit minimal variation. As the slurry progresses from initially entering to completely covering the detection zone, the integral values of the multiple echoes decrease significantly and eventually stabilize. Consequently, by analyzing the variation trend of the integral values of the multiple echoes, the position of the slurry relative to the probe’s detection zone can be effectively determined.

## 4. Experimental Validation and Results Analysis

This section begins by elucidating the physical significance of the key parameters characterizing ultrasonic wave propagation, namely, sound velocity, attenuation coefficient, and acoustic impedance. Subsequently, a comprehensive experimental platform was constructed. The platform primarily comprises a small multi-layer steel container, ultrasonic probes, an ultrasonic transmitter/receiver circuit board, a high-frequency data acquisition card, an industrial computer, and matched data acquisition and analysis software. Utilizing this platform, experimental investigations were conducted to study the propagation characteristics of ultrasound through the slurry, leading to the successful measurement of key parameters, including the propagation velocity, attenuation coefficient, and acoustic impedance of ultrasound within the slurry.

### 4.1. Experimental Platform Setup and Key Parameter Calibration

The fundamental physical properties of a material, such as its density and elastic modulus, can be characterized by its ultrasonic properties, primarily including the sound velocity, attenuation coefficient, and acoustic impedance. The relationship between these ultrasonic properties and the fundamental physical properties can be derived from the mathematical analysis of plane wave propagation within the material:

(13) $${\left(\frac{k}{\omega }\right)}^{2}=\frac{\rho }{E},$$

where *k* = *ω*/*c* + i*α* is the complex wavenumber of the material, *ω* is the angular frequency, *E* represents the elastic modulus, *ρ* denotes the material density, *c* is the sound velocity; and *α* is the attenuation coefficient.

The propagation velocity of ultrasound in a material depends on its fundamental physical properties. For low-attenuation materials (satisfying the condition *α* ≪ *ω*/*c*), the expression for the complex wavenumber can be simplified, yielding the following formula for the sound velocity *c*:

(14) $$c=\sqrt{\frac{E}{\rho }}.$$

The sound velocity in a material is primarily determined by its elastic modulus and density. In most cases, the variation in elastic modulus among different materials is considerably greater than the variation in their densities. Consequently, the elastic modulus generally has a more pronounced influence on the sound velocity than density does.

The phenomenon where the amplitude of an ultrasonic wave gradually decreases as it propagates through a material is termed acoustic attenuation. Attenuation primarily originates from two mechanisms: the absorption of the wave by the material and the scattering of the wave by the material’s internal structure. Absorption arises from the material’s viscosity, thermal conduction, boundary friction, and various relaxation phenomena, leading to the irreversible conversion of acoustic energy into other forms of energy, such as heat. Scattering is caused by inhomogeneities within the material, such as its grain structure, suspended particles, impurities, or bubbles. These inhomogeneities scatter the ultrasonic wave in multiple directions, resulting in a reduction in acoustic energy along the original propagation path. Consequently, the acoustic attenuation characteristics of a material reflect its microstructural features and molecular-level interactions. The attenuation behavior of ultrasound in a material is described by the following mathematical model:

(15) $$A=A_{0}e^{-\alpha x},$$

where *α* is the acoustic attenuation coefficient of the material, *A*_0_ represents the initial amplitude of the ultrasonic wave, and *A* denotes the current amplitude at a propagation distance *x*.

When an ultrasonic wave propagates across an interface between two different materials, the phenomena of reflection, refraction, and transmission occur. The intensity of the reflected wave is primarily determined by the difference in the acoustic impedance between the two materials. The acoustic impedance of a medium is defined as the product of its density and the speed of sound within it, as given by the following relation:

(16) Z=ρc.

### 4.2. Experimental Measurement of Propagation Characteristics Parameters

In this section, the key ultrasonic propagation parameters—namely, the sound velocity, attenuation coefficient, and acoustic impedance—of the slurry medium are experimentally determined. These parameters are essential for accurately modeling the acoustic behavior of the measurement system and for interpreting the echo signal variations associated with liquid-level changes. All measurements were conducted under controlled temperature conditions, with the slurry temperature maintained at 22 ± 1 °C throughout the experiments to ensure consistency and reproducibility.

The key experimental parameters were selected as follows. The container wall thicknesses of 8 mm and 20 mm represent typical values for industrial sealed vessels, allowing validation under different acoustic attenuation conditions. The transducer frequency (1 MHz) and diameter (20 mm) were determined from the acoustic field simulation in Section 2.4, which showed that 1 MHz provides adequate penetration in the highly attenuating slurry while maintaining reasonable directivity, and 20 mm balances near-field energy and positioning accuracy. The slurry density of 1.8 g/cm^3^ corresponds to the actual industrial formulation of interest, and its acoustic properties were measured as described in Section 4.2. The excitation voltage (24 V) and sampling frequency (2 MHz) were chosen to achieve sufficient signal-to-noise ratio and temporal resolution based on the specifications of the commercial ultrasonic board and DAQ card used.

#### 4.2.1. Experimental Platform Setup

**Figure 9:**
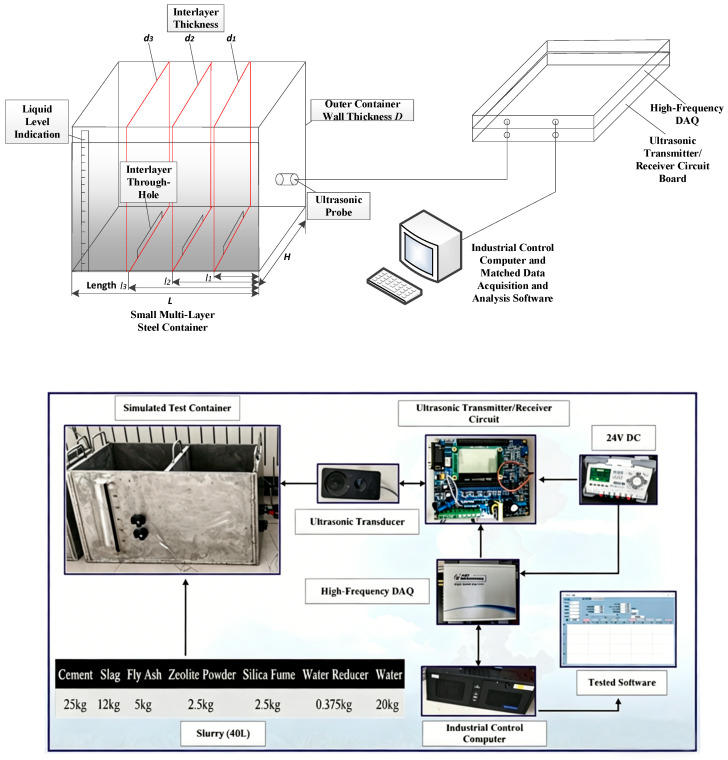
Technical specifications of the major equipment in the experimental system.

To measure the propagation characteristic parameters—namely, the sound velocity, acoustic attenuation coefficient, and acoustic impedance coefficient—of 1 MHz ultrasonic waves in the slurry material, a dedicated experimental system for testing ultrasonic propagation characteristics in slurries was designed and constructed. The core components of this system include a small multi-layer steel container, ultrasonic probes with a center frequency of 1 MHz, an ultrasonic transmitter/receiver circuit board, a high-frequency data acquisition card, an industrial control computer, and matched data acquisition and analysis software. The layout of the overall experimental system is shown in Figure 9.

The key technical specifications of the major equipment utilized in the experimental system are listed in Table 2.

#### 4.2.2. Sound Velocity Measurement

The propagation velocity of ultrasound in a material can be determined by measuring the reflected echo signals from two interfaces with a known separation. When the distance *H* between the two reflective interfaces is known, and the time difference *t* between the two received echo signals can be accurately measured, the ultrasonic propagation velocity *c* in the mixed medium is given by the following formula:

(17) $$c=\frac{2H}{t}.$$

**Figure 10:**
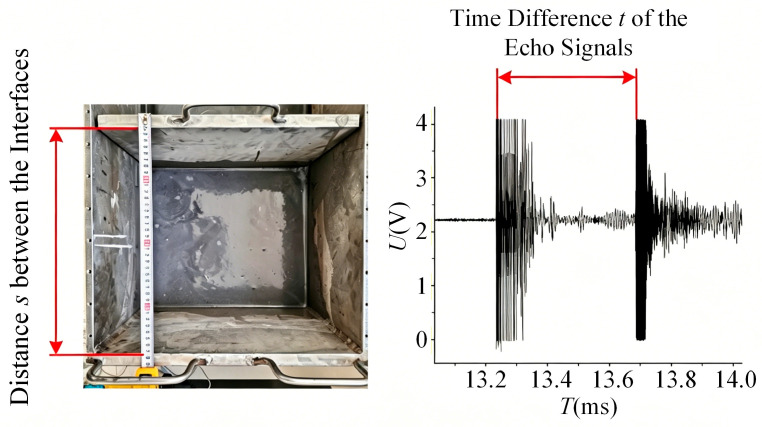
Schematic diagram of the key parameter measurement results for sound velocity.

Following the above principle, experimental measurements of the ultrasonic propagation velocity in the material were conducted. A representative result of the sound velocity measurement obtained using the experimental platform is shown in Figure 10.

Using the method described above, the time differences between echo signals at various interface distances were measured, and the experimental data are recorded in Table 3. The propagation velocity of ultrasound in the slurry was determined to be 2372 m/s through curve fitting of the experimental data.

#### 4.2.3. Attenuation Coefficient Measurement

Based on the ultrasonic attenuation law given by Equation (16), the formula for calculating the acoustic attenuation coefficient *α* can be derived as follows:

(18) $$\alpha =\frac{20}{2x}\mathrm{lg}\left(\frac{A_{0}}{A}\right).$$

**Figure 11:**
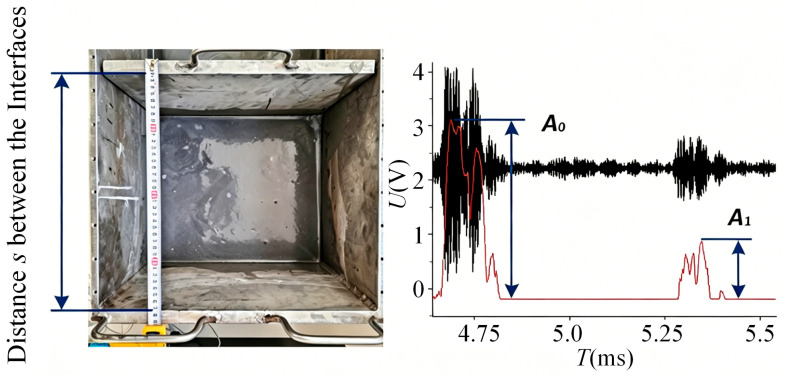
Schematic diagram of the key parameter measurement results for the attenuation characteristic.

When the propagation distance *x* of the ultrasonic wave is set to a known value, the attenuation coefficient *α* in the current medium can be calculated from the measured initial amplitude *A*_0_ and the final amplitude *A* at distance *x*, according to the attenuation model. A schematic diagram illustrating this measurement principle is shown in Figure 11.

Using the method described above, the ratio of the echo signal amplitude to the initial excitation signal amplitude was measured at different propagation distances, and the experimental data are listed in Table 4. The average attenuation coefficient of ultrasound in the slurry was determined to be 15.1 dB/m through curve fitting of the data.

#### 4.2.4. Acoustic Impedance Measurement

When an ultrasonic wave is incident normally on the interface formed by the container wall and a liquid medium (water or slurry), let *P*_0_ be the sound pressure amplitude of the incident wave and *P_r_* be that of the reflected wave. Denote the acoustic impedances of the container shell and the slurry by *Z*_1_ and *Z*_2_, respectively. Under normal incidence, the acoustic wave undergoes multiple reflections at the interface, generating a series of interface reflection echoes.

The peak sound pressure *P_n_* of the *n*-th reflection echo can be expressed as:

(19) $$P_{n}=P_{0}e^{-2n\alpha d}{\left(\frac{Z_{2}-Z_{1}}{Z_{2}+Z_{1}}\right)}^{n}R^{n-1},$$

where *d* is the thickness of the container shell, and *R* represents the sound pressure reflection coefficient at the interface between the ultrasonic probe and the outer container wall. Now, assuming all other experimental conditions remain unchanged, the container is filled with water (reference medium) and then with the mixed medium under test. Given that the acoustic impedance of water *Z*_2_, is a known standard value, the peak sound pressure *P_n_* of the *n*-th echo for the case filled with the test mixed medium can be expressed as:

(20) $$P_{n}^{\prime }=P_{0}e^{-2n\alpha d}{\left(\frac{Z_{2}^{\prime }-Z_{1}}{Z_{2}^{\prime }+Z_{1}}\right)}^{n}R^{n-1}.$$

Furthermore, the following relationship can be derived:

(21) $$\frac{P_{n}}{P_{n}^{\prime }}={\left(\frac{Z_{2}-Z_{1}}{Z_{2}+Z_{1}}\times \frac{Z_{2}^{\prime }+Z_{1}}{Z_{2}^{\prime }-Z_{1}}\right)}^{n}.$$

When *P_n_*, $P_{n}^{\prime }$, *Z*_1_, *Z*_2_ are all known, *Z*_2_ can be determined from the above relationship.

**Figure 12:**
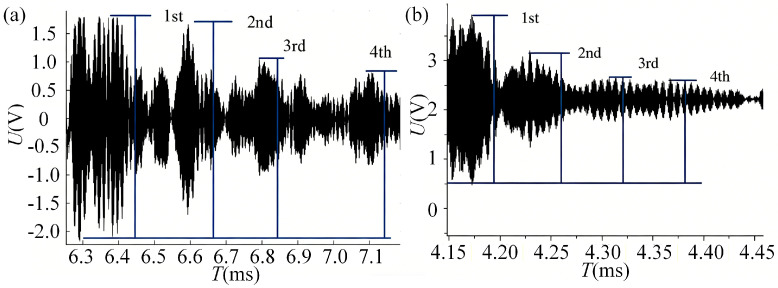
Measurement of ultrasonic impedance characteristics in the slurry ((**a**). water medium, (**b**). slurry medium).

The acoustic impedance of water *Z*_2_ is known to be 1.44 × 10^5^ g/(cm^2^ s), and the acoustic impedance of the container wall *Z*_1_ is 45.3 × 10^5^ g/(cm^2^ s). When the container was filled with water, the sound pressure value of the 4th echo was measured as *P*_4_ = 1.7 V, as shown in Figure 12a. When the container was filled with slurry, the corresponding value was measured as $P_{4}^{\prime }=0.64\;V$, as shown in Figure 12b. Using Equation (21), the acoustic impedance coefficient of the slurry *Z*_2_ was calculated to be 4.1 × 10^5^ g/(cm^2^ s).

### 4.3. Experimental Verification of Liquid-Level Interface Measurement

**Figure 13:**
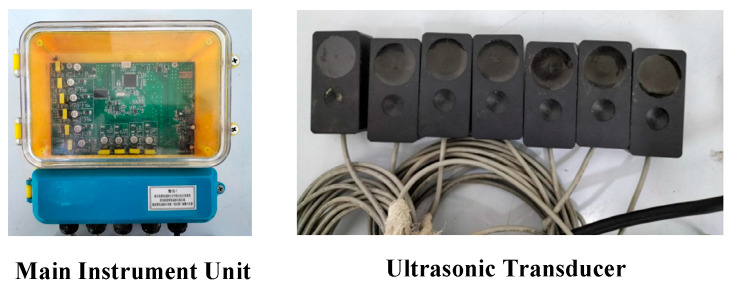
Multi-channel ultrasonic liquid-level interface measurement prototype system.

Based on the previously described principle for liquid-level interface detection in sealed containers, a multi-channel ultrasonic liquid-level interface measurement prototype system was developed as shown in Figure 13. A complete experimental test system was subsequently constructed based on this prototype to conduct accuracy tests for the liquid-level interface measurement.

The key technical specifications of the experimental test system is shown in Table 5.

**Figure 14:**
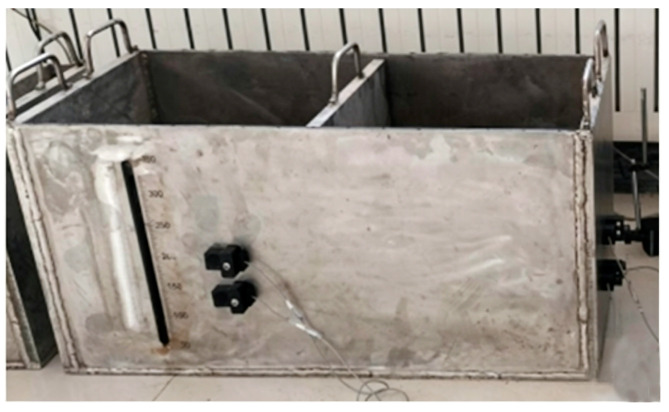
Installation positions of the ultrasonic transducers.

Ultrasonic transducers were installed on the outer surface of the simulated test container at the following positions: 10 cm and 17 cm heights on the container end face, and 13 cm and 20 cm heights on the container sidewall, as detailed in Figure 14. The host computer measurement software was then launched to record and save in real time the liquid-level interface echo signal measurements from these four transducer channels. Subsequently, the slurry was prepared according to the formulation specified in Table 5 and gradually poured into the simulated container until it was full. Finally, the acquired measurement data were analyzed and processed offline. Multiple transducers were installed at different heights to validate the feasibility of the method at various positions and under different wall thickness conditions. For applications requiring continuous level monitoring, a vertical array of such sensors can be implemented along the sidewall, which is a natural extension of this work.

**Figure 15:**
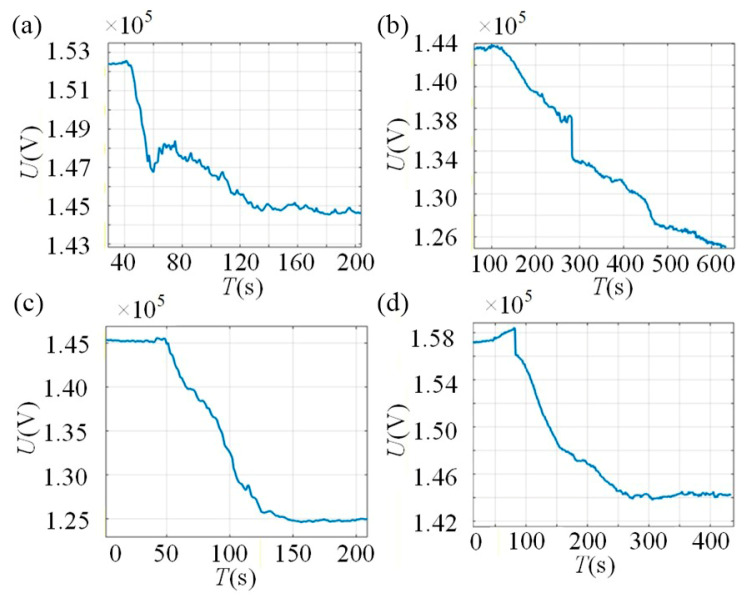
Variation in echo sound pressure during slurry injection: (**a**) Channel 1 (10.2 cm); (**b**) Channel 2 (16.8 cm); (**c**) Channel 3 (13.1 cm); (**d**) Channel 4 (20.2 cm).

The simulated container has an end wall thickness of 20 mm and a sidewall thickness of 8 mm. The transducers installed at the 10 cm and 17 cm heights on the container end face are designated as Channel 1 and Channel 2, respectively, while those installed at the 13 cm and 20 cm heights on the container sidewall are designated as Channel 3 and Channel 4, respectively. The variation in echo sound pressure received by these four channels during the slurry injection process is shown in Figure 15. The experimental results demonstrate that the proposed method effectively enables the monitoring of the slurry-filling level under both 8 mm and 20 mm wall thickness conditions.

As can be seen from Figure 15, when the injected slurry begins to enter the acoustic radiation area of an ultrasonic transducer, the echo sound pressure decreases significantly. As the slurry progressively covers the area completely, the echo sound pressure eventually stabilizes at a new level. The liquid level scale reading on the observation window at the moment the echo sound pressure starts to change significantly was recorded as the initial value, and the reading when the sound pressure stabilizes again was recorded as the final value. The difference between these two scale readings represents the liquid level change range to which the transducer channel responds. The corresponding data are listed in Table 6. The experimental results indicate that the measurement accuracy of the slurry liquid-level interface achieved with this method reaches ±2 mm.

## 5. Conclusions

This study has successfully developed and validated a novel method for liquid-level interface detection in multiphase mixed media based on ultrasonic transmission with a side-incidence configuration. The research systematically addressed the challenge through mathematical modeling, simulation, algorithmic development, and experimental verification. The principal findings and contributions are summarized as follows:

1. A comprehensive mathematical model of the entire ultrasonic measurement system was established. This model integrates the ultrasonic transmitter/receiver circuits, the wave propagation and multiple reflections within the multi-layer structure (container wall and medium), and the signal reception process. This model provides a theoretical foundation for system analysis and parameter optimization.

2. A dedicated detection algorithm was proposed. Based on the side-incidence approach, an echo sound pressure calculation model was constructed by analyzing the multiple reflections within the container wall. This led to the derivation of a formula for calculating the liquid-level height, forming the core algorithm for non-invasive interface localization.

3. The feasibility and accuracy of the proposed method were experimentally verified. The designed experimental platform enabled the successful measurement of key ultrasonic propagation parameters in the slurry: a sound velocity of approximately 2372 m/s, an attenuation coefficient of 15.1 dB/m, and an acoustic impedance of 4.1 × 10^5^ g/(cm^2^·s). Furthermore, liquid-level interface measurement tests under different wall thicknesses (8 mm and 20 mm) confirmed a measurement accuracy of ±2 mm, meeting the project requirements.

In conclusion, this research establishes that the side-incidence ultrasonic transmission method is a viable and accurate solution for non-invasive liquid-level interface detection in multiphase media within complex container structures. The integrated approach of modeling, simulation, and experimentation provides a solid foundation for practical application. However, the experimental validation was conducted under specific conditions: a steel container wall (8 mm and 20 mm thickness), a fixed slurry formulation (density 1.8 g/cm^3^), quiescent flow, and room temperature (22 ± 1 °C). The effects of other wall materials, larger thicknesses, elevated temperatures, turbulence, and composition variations have not been systematically evaluated and warrant further investigation. For continuous level monitoring, arranging multiple sensors in a vertical array along the sidewall would enable real-time tracking. Additionally, incorporating phase information could further enhance robustness and resolution. Future work will focus on algorithm robustness in high-noise environments, adaptive calibration for varying conditions, and extension to a wider range of media and industrial scenarios.

## Figures and Tables

**Table 1:** The initial values of the model parameters for the ultrasonic liquid-level interface measurement system.

System	Parameter	Value
Transducer	Crystal diameter (mm)	15
	Excitation frequency (MHz)	1
	Excitation voltage amplitude (V)	24
Container	Wall thickness (mm)	20
	Acoustic impedance coefficient (g/(cm^2^·s))	4.53 × 10^6^
Slurry	Sound propagation velocity (m/s)	2540
	Attenuation coefficient (dB/m)	17
	Density (g/cm^3^)	1.8
	Acoustic impedance coefficient (g/(cm^2^·s))	4.6 × 10^5^

**Table 2:** Key technical specifications of the major components in the experimental system.

No.	Component	Item	Parameter
1	Simulated Container	Length × Width × Height (mm)	800 × 400 × 400
		Sidewall Thickness (mm)	20
		Adjustable Interlayer Thickness (mm)	10–20
2	Transducer	Frequency (MHz)	1
		Crystal Diameter (mm)	20
		Excitation Voltage (V)	24
3	Ultrasonic Tx/Rx Board	Operating Frequency Range (Hz)	2 × 10^5^–5 × 10^6^
		Excitation Voltage Range (V)	0–200
		Maximum Signal Sampling Frequency (Hz)	2 × 10^6^
		Number of Channels	2
4	High-Speed DAQ Card	AD Resolution (bits)	14
		Maximum Sampling Rate (MS/s)	80
		Bandwidth (MHz)	40

**Table 3:** Ultrasonic propagation velocity values under different test conditions.

No.	Test Distance (m)	Time Difference of Echo Signals (ms)	Ultrasonic Velocity (m/s)
1	0.35	0.302	2330
2	0.45	0.372	2420
3	0.55	0.466	2360
4	0.65	0.546	2380

**Table 4:** Ultrasonic attenuation coefficient values under different test conditions.

No.	Test Distance (m)	Initial Excitation Amplitude/Echo Amplitude	Attenuation Coefficient (dB/m)
1	0.35	3.48	15.5
2	0.45	4.63	14.8
3	0.55	6.95	15.3
4	0.65	9.27	14.9

**Table 5:** The key technical specifications of the experimental test system.

No.	Item	Technical Specifications
1	Multi-channel Ultrasonic Prototype	(1) Power supply: 24 VDC (2) Output signal: RS485 standard protocol (3) 10 transceiver-integrated signal channels (4) Supported probe frequency: 1 MHz (5) Sampling frequency: 2 MHz (6) Single detection cycle: >250 ms (7) Echo signal calculation start time: 0–3 ms, adjustable
2	Ultrasonic Transducer	(1) Excitation voltage: 0–200 V (2) Excitation frequency: 1 MHz (3) Diameter: 20 mm
3	Test Slurry	(1) Volume: 60 L (2) Density: 1.8 g/cm^3^ (3) Attenuation coefficient: 15.1 dB/m

**Table 6:** Liquid-level interface detection results.

Probe Channel	Probe Center Position (cm)	Initial Sound Pressure	Initial Liquid Level	Stabilized Sound Pressure	Final Liquid Level (cm)	Mean Measured Level (cm)	Error (mm)
1	10	152,300	9.2	144,800	11.2	10.2	+2
2	17	143,800	15.8	125,200	17.8	16.8	−2
3	13	145,100	12.0	124,900	14.2	13.1	+1
4	20	158,100	19.2	144,200	21.2	20.2	+2

## Data Availability

Data will be made available on request.
